# Clinical, Epidemiologic and Ethical Aspects of Hysterectomy in Young Females With Intellectual Disability: A Multi-Centre Study of Public Hospitals in Mexico City

**DOI:** 10.3389/fpubh.2021.746399

**Published:** 2021-11-18

**Authors:** Horacio Márquez-González, Edith Valdez-Martínez, Miguel Bedolla

**Affiliations:** ^1^Department of Clinical Research, Federico Gomez Children's Hospital, Mexico City, Mexico; ^2^Congenital Heart Disease Department, Centro Medico Nacional Siglo XXI, Mexican Institute of Social Security, Cardiology Hospital, Mexico City, Mexico; ^3^Medical Research Unit in Clinical Epidemiology, Health Research Council of the Mexican Institute of Social Security, Mexico City, Mexico; ^4^Alamo Colleges-Northwest Vista College, San Antonio, TX, United States

**Keywords:** menstruation, hygiene, hysterectomy, intellectual disability, adolescent, epidemiologic studies, medical ethics, Mexico

## Abstract

**Background:** Non-therapeutic hysterectomy has been performed to this day in Mexican women with intellectual disabilities (IDs), but the rationale for performing the procedure has been rarely submitted to clinical ethics committees. The objectives of the present research were to determine the frequency of hysterectomy and the clinical and epidemiologic characteristics that associated to the indication of hysterectomy in girls and young females with IDs and to analyse the emerging ethical issues related to the procedure.

**Materials and Methods:** A medical chart review was conducted to identify female patients aged ≤ 25 years who had IDs based on anatomical pathologies and hospital records and underwent hysterectomy between January 2014 and December 2019 in nine high-concentration hospitals in Mexico City. Data were collected using a questionnaire developed *ex professo* and validated through a pilot study and analysed using the Statistical Package for the Social Sciences (SPSS) 21.0 software.

**Results:** Information of 234 female patients with or without ID who were ≤ 25 years of age was reported by the departments of anatomical pathology and paediatrics. Of the patients, 184 (79%) were excluded because the information reported was found to be erroneous or incomplete during the medical records review. Most of the 50 emales included in the study had moderate ID (*n* = 23, 46%) followed by those with severe ID (*n* = 17, 34%). The mean age at hysterectomy was 15 ± 2.9 years. Prophylactic-total abdominal hysterectomy was the most frequently performed (*n* = 42, 84%). A concurrence was observed between the parental and medical reasons justifying hysterectomy. The most frequent reasons were fertility control (parents vs. physicians: 46 vs. 42%), management of menstrual hygiene (28 vs. 30%) and risk of sexual abuse (6 vs. 6%).

**Conclusion:** This study showed that performing non-therapeutic hysterectomy is subject to the clinical judgement of physicians according to their perception of the patient's quality of life. Therefore, the ethical quality of the decision to perform the procedure in girls and young females resides in the ethical value of its consequences.

## Introduction

Through a secondary analysis of national representative data from the National Survey of Family Growth (NSFG) 2011–2015 in the United States, Li et al. (1) show that in the female civilian population whose ages ranged from 15 to 44 years, the prevalence rate of sterilisation using hysterectomy was higher among those with cognitive (22.1%, *n* = 272) and non-cognitive disabilities (24.7%, *n* = 150) than among those without disabilities (14.8%, *n* = 1,132). With adjustment for sociodemographic covariates, their logistic regression analysis yielded a significantly higher adjusted odds ratio (2.64, 95% confidence interval, 1.53–4.56, *p* < 0.001) for hysterectomy in the women with cognitive disabilities than in the other two groups (with other disabilities and without disabilities). Nevertheless, the NSFG excludes the institutionalised population, which could have more-serious disabilities and a different prevalence rate of hysterectomy.

A 2018 systematic review (2) of primary studies shows that most studies that included cases of non-therapeutic hysterectomy in women with IDs are based primarily on interviews with parents and carers and, to lesser extent, on medical records reviews and a case note review. Only one study included the self-report of women with ID (3). Parents and carers request the suppression or elimination of menstrual bleeding mainly because of their preoccupation with menstrual hygiene management and limited support networks. Meanwhile, the existing reports on medical-surgical experiences (2) are based on case series focused on the surgical technique and/or on short-term post-surgical morbidity. Physicians perceive hysterectomy as a safe procedure and the solution to menstrual cycle-related problems for women with IDs. In general, the more severe or profound the ID, the more physicians and parents/carers become interested in hysterectomy.

The findings of a systematic review published in 2019 (4) on the menstrual hygiene management requirements for disabled people are in agreement with another previous systematic review (2) and highlighted the barriers faced by carers and women with intellectual impairments, such as the lack of information and support provided to them, the lack of training in menstrual hygiene management and understanding of premenstrual symptoms experienced by the women concerned among carers and the high cost of menstrual products.

In a 2019 clinical literature review (5) related to contraceptive practises and reproductive health in women with IDs, the authors report that menstrual management is identified in multiple articles as a primary reason for which women with ID and their families approach physicians for reproductive health counselling, menstrual regulation or suppression and anticipatory treatment prior to menarche. Their findings also indicate that parents of women with IDs request sterilisation for their daughters owing to their reproductive health concerns, including challenges with menstrual management or lack of knowledge about other long-lasting, effective and reversible alternatives for pregnancy prevention.

The practise of non-therapeutic hysterectomy in women with IDs remains current despite the world-wide efforts to harmonise the rights of women with IDs with the Universal Declaration on Human Rights. This is probably a residue of the cumulative effect of two centuries (XIX and XX) of arguments to legitimise the ideas of eliminating the undesirable characteristics of the human race by preventing the reproduction of individuals with IDs, along with those with serious mental diseases and criminal behaviours (6). At present, this practise is considered discriminatory.

In Mexico, data from the national diagnosis on the situation of people with impairment (7) and the national measurement of poverty (8) show that the full exercise of the human and social rights is not assured for women with ID, the main causes are: low access to and poor quality of education; limited accessibility and availability of healthcare, and the fact that psychology and psychiatry services have never been a high priority at hospitals; to them must be added the high rate of people with impairment that live with an income below the poverty line. The main effects of these causes are reported to be: low human development and the consequent fact that women with ID depend on of their parents or family members, and high episodes of discrimination and violence against them. The problematization of reality implies that ID is not a simple condition, but the complex result of social reasoning. The IDs do not occur independently from their social context. In general, in the Mexican situation and in other Latin cultures, the prevalent values of a patriarchal culture along with other prevalent cultural beliefs and practises have perpetuated violence against, domination of, and other forms of coercion directed at women, and in particular at women with ID.

The Mexican health-care system is comprised of public and private components (9). Briefly, the public component consists of social security institutions and the institutions and programmes that cater to the population without social security. The former, financed by businesses, provides medical care to 63% of the national population. The latter, subsidised by the federal government, corresponds to the institutions belonging to the Secretary of Health. It provides medical care to the 35.5% of the population without social security. The private component caters to 2.8% of the population, especially middle-class citizens who prefer this type of care even if insured by the social security system. As for the classificatory system used to define the level of ID of the patient population, psychiatric services use the Diagnostic and Statistical Manual of Mental Disorders (DSM-5) as a guide, and the International Classification of Diseases (ICD-10) to define the classification of the IDs of patients.

The current and international ethical and legal guidelines (2) establish as the best practise that which is congruent with the best interest of women with IDs. Best practise is also non-discriminatory and promotes equity within the framework of health policies that prohibit obstetric violence, eradicate forced sterilisation and prescribe the creation of appropriate educational programmes for persons with IDs and their families. Nonetheless, pertinent Mexican laws do not define specific measures for women with IDs, although they are textually aligned with the recommendations of the international ethical guidelines for the care of people with disabilities (2). To understand the implications of this gap in knowledge, research such as that reported herein is necessary. The present research is the first Latin American study that addresses this issue.

The objectives of the present research were to determine the frequency of hysterectomy and the clinical and epidemiologic characteristics that associated to the indication of hysterectomy in girls and young females with IDs and to analyse the emerging ethical issues.

## Materials and Methods

### Design and Study Periods

A cross-sectional, descriptive, chart review study was conducted on women with IDs based on their anatomical pathologies and hospitals records from January 2014 to December 2019.

### Study Environment

The study was conducted in nine high-concentration hospitals in Mexico City, of which five were managed by the Secretary of Health and four by the Social Security System. These hospitals were selected because they are the principal national referral centres that provide medical care to female children and young women with IDs from various parts of Mexico. They have the accreditation, infrastructure and resources necessary for the genetic and/or gynaecologic care of female children and young women with IDs.

### Inclusion Criteria

All of the uterus of patients aged ≤ 25 years with evidence of ID as registered in their medical records were identified from the data bases of the Departments of Anatomical Pathological. Also, all diagnoses indicated for hysterectomy in patients with the same characteristics were identified in the databases of the departments of paediatrics and/or gynaecology.

### Exclusion Criteria

Female patients whose medical records were not found, those in whom the presence or absence of ID could not be determined, those whose ages were not registered or whose ages in the medical record did not coincide with that in the pathology report, were excluded from the study.

### Data Collection

Pilot tests were conducted to determine the face and content validity of the questionnaire developed *ex professo* for the data collection and to standardise the process for reviewing and analysing anatomical-pathological documentations and medical records.

The data were collected by two professional nurses using the questionnaire. The questionnaire included information about clinical characteristics such as aetiology and degree of ID; menstrual cycles; pre-hysterectomy gynaecologic diagnoses and treatment; clinical indication and type of surgical technique; and histopathological reports pertaining to the uterus of all the female patients included in the study. In addition, it included epidemiological characteristics of time, place and person, such as age, place of residence, education, occupation and legally responsible tutor.

The data collection process began with a review of the physical (notebooks) and electronic databases for the years 2014–2019 of the department of anatomical pathology. In these databases, the nurses searched for pathology reports and clinical summaries corresponding to hysterectomies performed in female patients with IDs of ≤ 25 years of age. Afterwards, the medical record of each patient was reviewed to complete and/or compare the information obtained. The search for cases was complemented with a review of the databases of the departments of paediatrics and gynaecology.

### Data Analysis

The data were analysed using the Statistical Package for the Social Sciences (IBM SPSS) version 21.0. Relative and absolute frequencies, and central and dispersion tendency measures were estimated. To identify differences between health institutions (Secretary of Health and Social Security System) as regard to the main epidemiological characteristics (i.e., degree and aetiology of ID, education and occupation), the chi-square or Fisher exact test (whichever was appropriate) was used. The Student *t* test was used to determine whether the mean age at the time of hysterectomy was statistically significantly different between the two sets of data. The Shapiro-Wilk test was performed as a test of normality (with *p*-value > 0.05) which corroborated a parametric distribution.

### Ethical Considerations

The research project was evaluated and approved by the research ethics committee of each participating hospital. The letter of approval of the research ethics committee along with an invitation to participate, which included an explanation of the study, were sent to the chairpersons of the departments of anatomical pathology, paediatrics, gynaecology and medical records archive to gain access to their physical and electronic databases, documents and medical records. Owing to the retrolective nature of the study, personal data (i.e., name and medical record number) and sensitive data (i.e., data related to the patient's medical and personal histories) were subjected to a dissociation procedure to protect their identities and privacies during and after the research.

## Results

During the 6 year study period, 234 female patients aged ≤ 25 years underwent hysterectomy, of whom 50 were included in the study and 184 (79%) were excluded. The reasons for exclusion were lack of information about the diagnosis of ID (*n* = 127, 69%), lack of medical records (*n* = 42, 23%) and discrepancies in the name and/or age between the anatomopathological report and medical record (*n* = 15, 8%). Of the 50 patients included, 36 (72%) were from hospitals belonging to the Secretary of Health and 14 (28%) were from hospitals under the Social Security System.

### Demographics

Table 1 lists the distribution of the frequencies of the socio-demographics characteristics of the female patients included in the study.

**Table 1:** Demographic characteristics of the 50 girls and young female with ID and hysterectomy.

	**Number (%)**
**Institution/hospital**	
Secretary of Health/A	36 (72)
Social Security System/B	4 (8)
Social Security System/C	4 (8)
Social Security System/D	6 (12)
**Education**	
Primaria	8 (16)
Secondary	4 (8)
MAC*	22 (44)
Uneducated	11 (22)
Missing data	5 (10)
**Occupation**	
No occupation	22 (44)
Student	18 (36)
Domestic work	2 (4)
Gainful occupation	1 (2)
Missing data	7 (14)
**Legally responsible tutor**	
Mother	28 (56)
Both parents	16 (32)
Grandmother	2 (4)
Aunt	1 (2)
Missing data	3 (6)

*MAC, “Multiple Attention Centre” earlier known as “school of special education.” ID, Intellectual disability.

Of the 50 women with IDs, 22 (44%) attended “multiple attention centres,” earlier known as schools of special education, 8 (16%) attended primary schools, 4 (8%) attended secondary schools and 11 (22%) were out of school. No statistically significant differences (*p* = 0.1) were found between the health institutions (Secretary of Health and Social Security System). Of the patients, 22 (44%) had no occupation, 18 (36%) were students and 2 (4%) worked as domestic workers in their own home. Only one patient had a gainful occupation. The highest proportion of women without any occupation or with domestic work was observed in the group treated in the hospitals belonging to the Secretary of Health (*p* = 0.02). The legal representative was the mother in 28 patients (56%), both parents in 16 (32%) and a grandmother or an aunt in three (6%).

### Clinical Epidemiologic Characteristics

As shown in Table 2, the mean ages at the times of hysterectomy and menarche were 15 ± 2.9 and 12 ± 1.6 years, respectively. The youngest patients at the time of hysterectomy were those from the hospitals of the Secretary of Health (*p* = 0.001).

**Table 2:** Clinical epidemiologic characteristics of the 50 girls and young female with ID and hysterectomy.

	**Number (%)**
Women without menarche	4 (8)
Age at menarche in years, average (DS)*	12 (±1.6)
Age at hysterectomy in years, average (DS)*	15 (±2.9)
**ID degree**	
Severe and profound	17 (34)
Moderate	23 (46)
Superficial and mild	6 (12)
Unspecific	4 (8)
**Aetiology of the ID**	
Genetic-primary	17 (34)
Multi-factorial	33 (66)
**Pregnancy**	
Nulliparous	48 (96)
Missing data	2 (4)
**Types of hysterectomy**	
“Prophylactic”-total abdominal hysterectomy	42 (84)
Total laparoscopic hysterectomy	6 (12)
Subtotal abdominal hysterectomy	1 (2)
Missing data	1 (2)
**Pathology reports**	
Normal	11 (22)
Didelphic	1 (2)
Bicorned	1 (2)
Uterine myoma	1 (2)
Missing data	36 (72)
**Immediate and mediate postoperative complications**	
No complications	30 (60)
Fever	4 (8)
Abdominal wall abscess	3 (6)
Urinary retention	1 (2)
Missing data	12 (24)

ID, Intellectual Disability. ^*^Shapiro-Wilk test with p-value > 0.05.

The most frequent degrees of ID were moderate (*n* = 23, 46%) and severe or profound (*n* = 17 patients, 34%). The patients with moderate IDs were most frequent in the hospitals belonging to the Secretary of Health, whereas those with severe or profound IDs were most frequent in the hospitals belonging to the Social Security System (*p* = 0.002).

The aetiology of the ID was multi-factorial in 33 (66%) of the 50 patients, including hypoxic-ischaemic encephalopathy (*n* = 14), tuberous sclerosis (*n* = 2), anaplastic ependymoma (n = 1) and multi-factorial disorder (*n* = 16).

In the remaining 17 (34%) patients, the aetiology was classified as genetic-primary, including Down syndrome (*n* = 8) and 18p *de novo* deletion (**n** = 3). The rest of the genetic diagnoses, namely Williams syndrome, Stickler syndrome, McKusick-Kaufman syndrome, Trisomy 17 and 22q11 and q22-23 deletions, occurred in one patient each. No statistically significant differences (*p* = 0.3) were found between the participating health institutions.

Of the 50 patients with ID, 48 (96%) were reported as nulliparous and two had unknown histories of pregnancy in their medical records. “Prophylactic”-total abdominal hysterectomy was the most frequently performed (n = 42, 84%), followed by “total laparoscopic hysterectomy” (*n* = 6, 12%). Of the 50 patients, 36 (72%) had no histopathological reports. The pathology reports of the 14 remaining patients indicated normal (*n* =11, 22%), didelphic (*n* = 1, 2%), bicorned (*n* = 1, 2%) and uterine myoma (*n* = 1, 2%).

Trans-surgical complications occurred in four patients, of whom three showed a dehiscence of the vaginal cuspid and one had an anaphylactic reaction. Immediate postoperative complications of hysterectomy occurred in eight women, of whom four had fever, three had an abdominal wall abscess and one had urinary retention.

**Figure 1:**
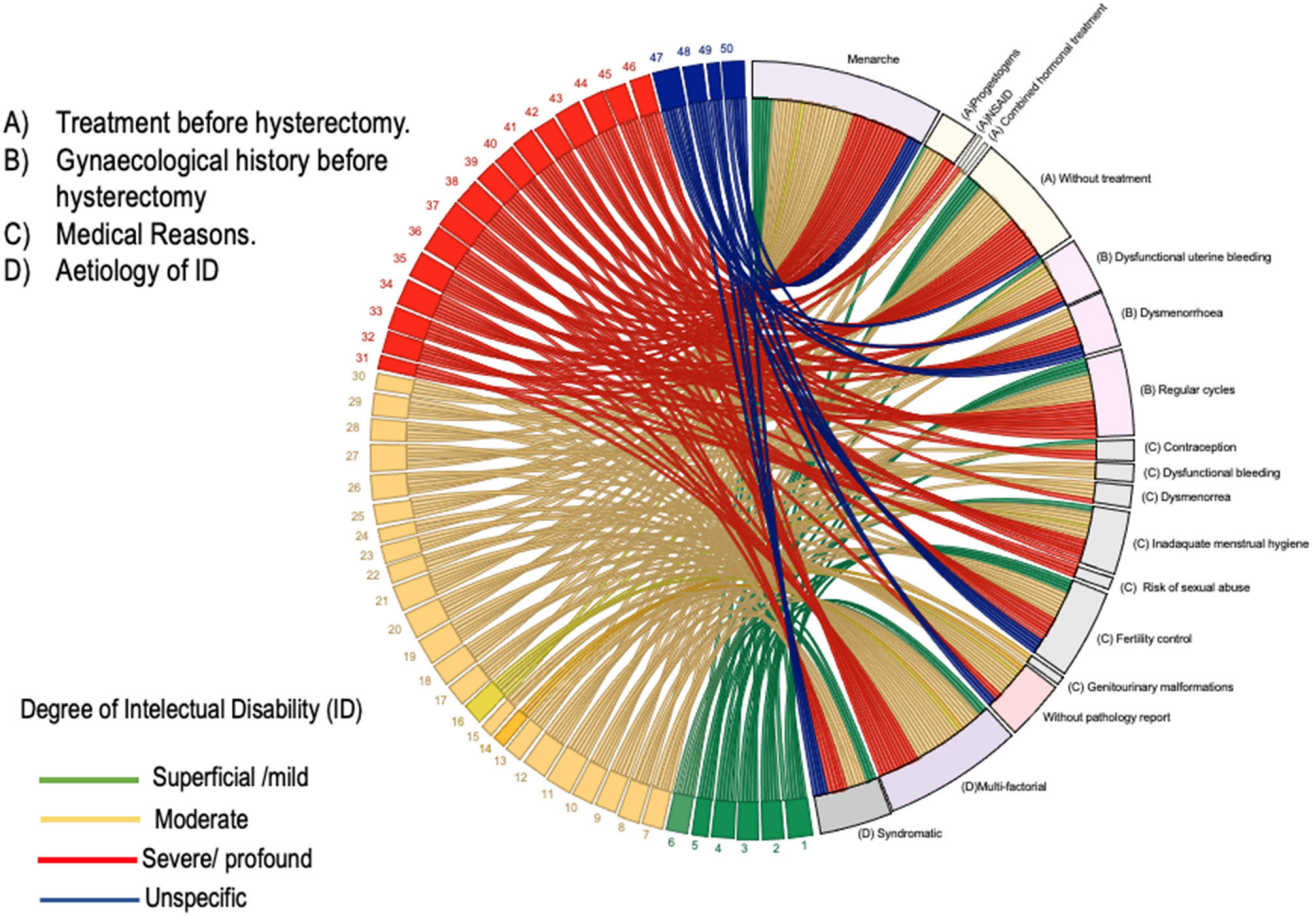
A chordal graph of clinical characteristics of the 50 girls and young females with ID. Explanatory footnote. On the numbered (left) half of the chordal graph, the numbered bands represent each one of the participating women. Each woman's band is released towards the unnumbered (right) half of the graph. The ending of bands on the unnumbered side of the graph represent one of the variables of interest for that woman, e.g., the medical reasons for the hysterectomy, the aetiology of ID, and the gynaecological history and the gynaecological treatments before hysterectomy.

The reasons for the hysterectomy given by the parents/carers of the patients with IDs and the physician's rationale coincided in almost all the cases (Table 3). The most frequent reasons that justified the hysterectomy included fertility control (46% of the parents and 42% of the physicians) and inadequate menstrual hygiene (28% of the parents and 30% of the physicians), followed by risk of sexual abuse (6% of the parents and 6% of the physicians), dysmenorrhoea (6% of the parents and 10% of the physicians), dysfunctional uterine bleeding (4% of the parents and 8% of the physicians), genitourinary malformations (4% of the parents and 4% of the physicians) and personality changes before menstruation (6% of the parents). Within the mentioned distribution of frequencies are four women who had not experienced the onset of menstruation (menarche) and underwent “prophylactic hysterectomy” on the recommendation of the treating gynaecologist. Two of them were classified as having moderate ID; and the other two, severe and profound ID. The physician's reasons for indicating prophylactic hysterectomy were to avoid sexual abuse (*n* = 3), to control fertility (*n* = 3), and for menstrual hygiene management, which the physician judged to be impossible for the patient to perform by herself (*n* = 1) Figure 1. None of these cases was presented to a clinical ethics committee for evaluation.

**Table 3:** Reasons that justified the hysterectomy.

	**Parents**	**Physicians**
	**Number (%)**	**Number (%)**
Inadequate menstrual hygiene	14 (28)	15 (30)
Fertility control	23 (46)	21 (42)
Risk of sexual abuse	3 (6)	3 (6)
Dysmenorrhoea	3 (6)	5 (10)
Dysfunctional uterine bleeding	2 (4)	4 (8)
Genitourinary malformations	2 (4)	2 (4)
Personality changes before menstruation	3 (6)	-

### Pre-hysterectomy Clinical Characteristics

Table 4 shows the frequency distribution of the durations of bleeding, menstrual cycles and gynaecologic treatments before hysterectomy. More than 50% of the women had regular menstrual cycles and bleeding that lasted ≤ 8 days. Hormonal treatment (progestogen monotherapy or combined oestrogen-progestogen) was used as a contraceptive method (*n* = 5), for the management of dysfunctional uterine bleeding and/or dysmenorrhoea (*n* = 4) and for menstrual hygiene management (*n* = 1). Treatment with non-steroidal anti-inflammatory drugs was used to manage dysmenorrhoea (*n* = 1) Figure 1.

**Table 4:** Pre-hysterectomy clinical characteristics.

	**Number (%)**
**Duration of bleeding (days)**	
≤ 5	22 (44)
6–8	9 (18)
9	1 (2)
15	1 (2)
Missing data	13 (26)
No menarche	4 (8)
**Menstrual cycles**	
Regular menstrual cycles	32 (64)
Dysfunctional uterine bleeding and/or dysmenorrhoea	21 (42)
Missing data	1 (2)
No menarche	4 (8)
**Gynaecologic treatments before hysterectomy**	
Progestogen monotherapy	9 (18)
Combined oestrogen-progestogen	1 (2)
NSAIDs^*^	1 (2)
Missing data	6 (12)

* *NSAIDs, Non-steroidal anti-inflammatory drugs*.

## Discussion

The results of our study suggest that in Mexico, non-therapeutic hysterectomy continues to be one of the most common procedures performed in females with ID. The reasons described in the medical records for performing hysterectomy were based on the benefit that hysterectomy represents for females with ID, especially those with moderate, severe and profound IDs. The reasons were to control fertility, solve problems with menstrual hygiene and protect from the risk of sexual abuse. Moreover, it was the first-choice treatment for the management of dysfunctional uterine bleeding and/or dysmenorrhoea.

This practise raises fundamental ethical questions such as the following: does the performance of non-therapeutic hysterectomy in women with ID go against the main fiduciary obligation of the physician, which is to provide due care? Is the performance of non-therapeutic hysterectomy in women with moderate or severe ID an act of beneficence? In deciding whether to perform hysterectomy in women with IDs, is the safety of the surgical procedure the only thing that matters? Are all the interests that must be represented during the decision-making process adequately represented when a non-therapeutic hysterectomy is chosen as the treatment? These questions result in four separate but complementary lines of arguments that deserve discussion.

First, while the results of the study show that the parents were in accord with the physicians in the decision to perform hysterectomy and in the reasons that justify it, and no evidence was found in the medical records of any type of undue influence to obtain the informed consent required, ethical issues about the appropriateness of the decision may even arise. On the one hand, the respectful acceptance of medical recommendations is part of the value of courtesy and deference to authority that is characteristic of the Mexican culture (and other countries with similar cultures), where directly contradicting a physician is considered disrespectful and discourteous (10). Furthermore, it is also the health literacy level of the parent/carer (11), which is related to their personal characteristics and the social resources needed to understand and use medical information to make decisions regarding their daughter's health. Thus, the lower the order of competencies or abilities to critically appraise the medical information received, the lesser the chance of parent/carer involvement in medical decision making (11). Consequently, parents/carers may assume a “passive patient” role. On the other hand, even when a parent/carer participates actively in medical decision making, their election will depend to a large extent on the type and amount of information presented by the treating physician and the terms in which the information is disclosed (12). For example, the fact that hysterectomy is presented as a safe surgical procedure and having preventive benefits (i.e., to avoid pregnancy, eliminate menstrual bleeding and avoid the risk of sexual abuse) will influence positively the parent's/carer's choice. Despite that hysterectomy becomes extremely attractive, the manner of framing information is misleading because hysterectomy does not protect women with IDs from the risk of sexual abuse. This way of framing information affects understanding and voluntariness; thereby, parents/carers are denied of a truly autonomous choice.

Second, hysterectomy must not be performed arbitrarily, that is, based on simple matters of opinion, preferences or partiality of physicians. An ethical problem emerges when physicians decide on a treatment approach without a clear view of the facts or without considering the interests of all persons involved and without the prudent use of the available empirical research. This study was primarily descriptive, so we could not elaborate on how the participating physician's clinical judgements were developed. Nonetheless, the medical reasons indicated in the medical record, based on which the decision and recommendation to perform hysterectomy was made, reflect the interests (values and objectives) of the treating physicians: whether they behave as activists in favour of hysterectomy, disapprove the lifestyles of the women in question or demonstrate their gender prejudice. From an ethical point of view, Lain-Entralgo (13) and Campbell (14), among others, point out that physicians act ethically only when they are aware of their feelings and values and other factors that can mislead their reasoning process.

Third, even if physicians (gynaecologists in particular) consider it their obligation “not to cause harm” to women with IDs, the fact that they preferred to indicate non-therapeutic hysterectomy for those with moderate, severe and profound IDs, even pre-menarche, and as the treatment of first election for dysmenorrhoea and/or dysfunctional uterine bleeding seemed to depend more on the importance they assign to the physical, mental and social situation of women with ID. When beneficence is technically conceived and inappropriately interpreted, it entails an illegitimate use of power that undermines women's rights. Non-maleficence requires that the recommendation or indication be based on a continuous and forthright dialogue between the treating physician and the parents/carers (and women with IDs, if possible) (13). Dialogue without undue influences, during which the treating physician attempts to elucidate both the parents/carer's values and objectives and the personal and social realities of the women and their families; as well as, helping them to understand, with truly reasons, the benefits and risks of hysterectomy so that they can to make a more reasoned decision. Accordingly, even though beneficence is the primary and most fundamental reason that dictates a medical action; non-maleficence, respect for autonomy and justice set the limits to such actions.

Fourth, the right to health is a concrete, not abstract, natural right, which must be enjoyed by women with IDs in their context (2). The fact that physicians may have the intention to protect their female patients with ID (by performing a hysterectomy) against threatening individuals and social disadvantages does not provide an adequate basis for asserting the physicians are acting with justice. Physicians themselves must be aware and judge whether their intention is discriminatory and will have a negative impact in the long term on the health of women with IDs (15). Moreover, physicians must understand that human rights are a rational system of human relations where beneficence and compassion are essential components of respect for the autonomy of parents/carers (and women with IDs, if applicable), which can be destroyed (in women with IDs) by piety and condescension.

### Strengths and Limitations

This is the first study of its kind in Latin America. It is also the first national study that included data from the main reference centres for girls and young females with IDs. It also analysed the largest data set in the last decade. The methodological rigour with which it was conducted included the collaboration of expert nurses devoted exclusively to the search, collection and codification of information.

Nevertheless, the study has limited value for the estimation of the frequency of non-therapeutic hysterectomy cases because of the under-registration of the surgical procedure, especially in women with IDs. Of the nine hospitals that participated in the study, only three had registered the uterus of women with IDs in the department of anatomical pathology. In one of the participating hospitals, women with IDs were listed in a physical database (notebooks) of the department of paediatrics. In the other five hospitals (four belonging to the Secretary of Health and one belonging to the Social Security System), despite the fact that the gynaecologists reported the performance of hysterectomies in women with ID (verbal communication), they were not registered in the department of anatomical pathology or paediatrics and/or gynaecology. Most uteri were not registered in the department of anatomical pathology because they were judged as healthy by the performing surgeon and thus not sent to the department of anatomical pathology (verbal communication).

Under-registration also explains why ID is not considered a primary diagnosis and is difficult to identify in hospital registries. It also explains the incomplete or contradictory information between several pathology reports and medical records. In some cases, no medical record was found. All these are a reflection of in real life.

Although the scenario was reconstructed with data from two sources, namely pathology reports and medical records, it is highly probable that they did not described in detail the discussions between physicians and parents/carers/women with IDs. However, the fact that the participating hospitals belonging the Secretary of Health (financed by the federal government and provide medical care to individuals without social security) showed higher frequency rates of non-therapeutic hysterectomy in women aged ≤ 15 years who had moderate IDs and no occupation might reflect the presence of a social gradient in health outcomes associated with the unfair distribution of the social determinants of health. This assertion is consistent with those reported in other similar studies in developing countries (2).

Despite the limitations, this study is particularly useful and has clinical and social value because it underlines the urgency of incorporating ethical thinking into quotidian clinical practise. The study shows that there is an urgent need to reformulate strategies and statutes in this area of public health. It shows it by describing the clinical epidemiological characteristics of the study population and identifying the reasons for prescribing and subjecting its members to a hysterectomy.

The study suggests the need for further clinical and socio-medical research to examine in depth the contextual and semantic factors that influence the perceptions of parents/carers and patients themselves and their impacts on the treatment decisions pertaining to these patients. Moreover, this study reveals that epidemiologic studies must be conducted in private institutions where the frequency of non-therapeutic hysterectomy is not known. Clinical studies are also needed to evaluate the long-term physio-pathological impact of non-therapeutic hysterectomy on women with IDs.

In conclusion, the results of our study show that non-therapeutic hysterectomy continues to be performed for women with IDs in Mexico to this day and, in many cases, without the involvement of the clinical ethics committees of the respective hospitals. Our results also indicate the need to determine the quality of life of women with IDs without arbitrary judgements based on personal interests and social values attributed to women with IDs. It becomes, then, crucial to recognise that ethical quality of medical decisions and interventions should and ought to be based on the self-awareness and self-understanding of physicians and all other persons participating in the decision-making process.

## Data Availability Statement

The data supporting the conclusions of this article are available from the corresponding author on reasonable request.

## Ethics Statement

The research project was evaluated and approved by the Research Ethics Committee of each participating hospital. Written informed consent from the participant's legal guardian/next of kin was not required to participate in this study in accordance with the national legislation and the institutional requirements.

## Author Contributions

HM-G contributed to the formulation of the research question and design, contributed to the data collection, participated in the data analysis and interpretation, drafted the initial versions of the manuscript, and critically reviewed and approved the final manuscript as submitted. EV-M was responsible for the research questions and design, coordinated the data collection, supervised the study, participated in the data entry, analysis and interpretation, critically reviewed the initial versions of the manuscript, and wrote the final manuscript as submitted. MB revised the manuscript for critical intellectual content and translated it into English. All authors have read and approved the final version of the manuscript.

## Funding

The funds received for open access publication fees are from the Research Department of the Hospital Infantil de Mexico Federico Gómez (Federico Gomez Child's Hospital).

## Conflict of Interest

The authors declare that the research was conducted in the absence of any commercial or financial relationships that could be construed as a potential conflict of interest.

## Publisher's Note

All claims expressed in this article are solely those of the authors and do not necessarily represent those of their affiliated organizations, or those of the publisher, the editors and the reviewers. Any product that may be evaluated in this article, or claim that may be made by its manufacturer, is not guaranteed or endorsed by the publisher.
